# An Interesting Outbreak of Typhoid Fever

**Published:** 1908-03-07

**Authors:** 


### Public Health and Hygiene.

### AN INTERESTING OUTBREAK OF TYPHOID FEVER.

The path of medical investigation is strewn with the most cunningly devised pitfalls, and he must walk warily indeed who would escape falling precipitously even where the ground seems surest. It is just when the puzzling facts of a painstaking inquiry are resolved in the restful harmony of a diagnosis or the satisfying transparency of a not too hasty theory that a healthy scepticism should be most alert. It is a feat not often attained, requiring courage, discipline, and active and unslumbering watchfulness, to shatter the plausible or "pretty" explanation on which one has happened in disentangling an intricate problem.

We are moved to make these observations by a report we have read with great interest made by Professor Matthew Hay, Medical Officer of Health of Aberdeen, on an epidemic of typhoid fever in Peterhead during 1907.

### The Case for Water Contamination.

The epidemic, referred to in our issue of September 14, commenced in a small but sudden outbreak, the first cases of which were notified early in June of last year. "Owing to the apparently general character of the outbreak, the water supply was properly at once suspected as the source of the infection, the more that it was well known that the conditions under which it was collected were at several points unsatisfactory, as was evidenced by the fact that the Town Council had for some time been discussing a scheme for improving and increasing the water supply." On June 8 the analyst, Mr. Tocher, reported that the water in the reservoirs was of bad quality, and that in the case of one of the small sources of supply it was "quite evidently surface water" and that "the sample is a dilute sewage and its use may lead to serious consequences."

Prior to June 8 only one case of typhoid fever had been notified during the month, but on the 8th twelve cases were notified, and daily notifications followed almost without intermission until the end of August. Sporadic cases continued to be notified until the end of October.

### Confirmatory Evidence.

The water at the beginning of the epidemic was at various points open to pollution. There was no provision for filtering it. The danger was increased by the fact that, as the gathering ground of the water supplies was at no great distance from the town, several farmers within the gathering ground were in the habit of manuring their fields in part with town's manure or refuse. There were in the town a number of old-fashioned, badly kept privies connected with ash-pits, so that the town's refuse must have consisted in part of the contents of privy middens. There was no doubt, both from an inspection of the wells or springs themselves, and from chemical and bacteriological analysis, that the water was "largely of the nature of ordinary field drain or sub-soil water." Little wonder, under these circumstances, that the water was suspected as the cause of the outbreak. This view was further confirmed by the fact that in his Majesty's convict prison, situated about two miles out of the town, but possessed of its own separate water supply, not a single case of the disease had arisen. Moreover, after cutting off from the town's water supply the graver sources of pollution, notifying the inhabitants publicly and individually of the great danger of using unboiled water for domestic purposes, and supplying filtered water from the Admiralty Works, the epidemic began to decline.

It might in these circumstances have been thought unnecessary to probe further into the matter. The outbreak read like a book, and was to all appearance an ordinary commonplace example of water-borne typhoid fever due to the usual neglect of simple sanitary precautions. In an interim report of the medical officer of the Scottish Local Government Board it was stated that the cases were not associated with any particular milk supply, and milk was therefore excluded as the cause of the spread of the disease. It is a feature of most epidemics that the earliest cases notified are rarely the earliest cases occurring. In the light of subsequent events the candid practitioner reviews the provisional diagnosis, which is all that usually can be made with the limited data on which he must act.

### Doubts Arise.

Dr. Hay says: "I had learned from the medical officer of health that many of the earliest cases, owing to some doubt as to their exact character at the time, had remained unnotified, as so frequently happens in unexpected outbreaks of such a vaguely defined disease as typhoid, and I accordingly asked him to procure for me, if possible, a list of such cases from the various medical practitioners."

As a result of the courteous response of the medical practitioners, information was elicited, without which it would have been "impossible to form any clear opinion as to the origin of the outbreak." In consequence of this information Dr. Hay came to believe that the epidemic began about the commencement of the second week of May. In the whole of the preceding months of the year only one case of typhoid had been notified—namely, on April 6, the illness having begun on March 16. The new cases discovered were investigated in relation to the milk supply, Dr. Hay, early in September, having found himself "drifting towards the opinion that the balance of the evidence was against the epidemic having originated in contamination of the water supply."

### Exoneration of the Water-Supply.

Doubt as to the truth of the original explanation of the outbreak was raised by a detailed analysis of the cases in relation to the distribution of the town's water coming from two different sources. It was found that the cases were distributed quite irrespective of the distribution of the water coming from these distinct and separate sources.

If the epidemic originated in typhoidal contamination of the water, it was exceedingly unlikely that both water supplies became contaminated at the same time; yet such an assumption was necessary to account for the distribution of the earlier cases of the epidemic.

### The Milk Supply Suspected.

The investigation of the earlier unnotified cases modified the view, based upon investigation of the notified cases only, that there was no association of the cases with any particular milk supply. It was now discovered that out of seven persons who became ill during the first week, six were supplied wholly or in part from one dairy farm; while of the twenty-four cases of the second week, twenty-one were supplied by the same farmer. In the third week he supplied with milk twenty out of thirty-three cases. During the first three weeks of the epidemic every case of typhoid, with one possible exception, occurred in streets visited by this farmer in selling his milk. Twenty-four streets or lanes were invaded by the disease in these weeks. In nineteen of them the first known case in the street was a customer either of this farmer or of a dealer receiving his milk. A remarkable succession of illnesses in connection with this farm was now brought to light.

### The Clues Followed Out.

Between the middle of May and July 24 no fewer than seven persons associated with the farm were successively attacked by illnesses, the symptoms of which were those of typhoid fever, and two of them were notified on June 27 and 28 respectively as suffering from this disease. Whether the first of these cases was typhoid or not, however, it did not occur sufficiently early to account for contamination of the milk, which, it was now inferred, produced its first cases in the town from May 8 onwards. The date of contamination could scarcely have been later than the beginning of May or the end of April. These cases at the farm were probably therefore the result of the infection of the milk, and not the cause of it. "I had got no further than this after my first visit to Lochside Farm," says Dr. Hay, "when, as I believed, I had obtained all available information as to possible sources of infection. At a second visit, however, made some weeks later, I learned what I had failed to discover previously—that the maidservant, who had been employed at the farm for about three years, and was there during the past summer, had gone home to her parents in Peterhead in December 1906 to nurse her mother and other members of the family who were seriously ill, and that she did not resume her employment at the farm until April 28 or 29. . . . I found that every member of the family ordinarily resident in the house had, since the beginning of the preceding November, been laid down with febrile illness, accompanied by more or less of diarrhœa. The first to be affected was the father, who was three weeks in bed, and very feeble for two or three weeks afterwards. He suffered much from fever and lost flesh. His illness was followed by that of two young daughters, one of whom lay in bed for nearly two weeks and the other for ten weeks. Then the mother took ill about the beginning of December, and was confined to bed for fourteen weeks. The only remaining member of the household was an imbecile boy, who also took ill with what his medical attendant considered, and still considers, to be influenza with diarrhœa, and died on December 26, after five days' illness. All the others recovered. The blood and urine of each member of the family were now"—after Dr. Hay's second visit—"submitted for bacteriological examination."

### The Decision Reversed.

Widal's reaction was uniformly negative, but the urine of the mother yielded the bacilli of typhoid. "The organism gave all the cultural tests characteristic of typhoid, was agglutinated with a high dilution of typhoid serum, and was rapidly pathogenic to lower animals." There could therefore be no doubt as to the nature of the mother's illness or that she was infectious right up to the time when her daughter left her to return to the farm on April 28 or 29, when she at once began to take part in the work of the dairy. Nine to ten days after the return of the servant to the farm the outbreak of typhoid began among the customers of the dairy. The chain of evidence as to the cause of the outbreak was now so complete as to leave no doubt as to its immediate origin.

"In my opinion," adds Professor Hay, "the epidemic in Peterhead, after being started by milk infection, which planted a large number of cases throughout the towns, was kept going mainly, and perhaps wholly, by contact infection."

The report raises many points of interest. Most of all, it shows the importance of pursuing investigation after a general explanation of the facts consonant with accepted views offers a tempting *prima facie* solution of the problem. It exhibits the need of going behind the data furnished by the statutory notifications and of pushing inquiry into suspicious illnesses, the true character of which is frequently only revealed in the light of epidemiological as distinguished from clinical observation. It shows how, in the presence of a contaminated water supply, an epidemic of typhoid simulating a water-borne outbreak may be due to quite other causes which a cursory investigation seemed to negative.

It affords a further illustration of the fact, for so long disputed, that typhoid fever is a disease which spreads by contact infection to an extent not yet sufficiently recognised.

